# The miR-24-Bim pathway promotes tumor growth and angiogenesis in pancreatic carcinoma

**DOI:** 10.18632/oncotarget.6257

**Published:** 2015-10-28

**Authors:** Rui Liu, Haiyang Zhang, Xia Wang, Likun Zhou, Hongli Li, Ting Deng, Yanjun Qu, Jingjing Duan, Ming Bai, Shaohua Ge, Tao Ning, Le Zhang, Dingzhi Huang, Yi Ba

**Affiliations:** ^1^ Department of Gastrointestinal Oncology, Tianjin Medical University Cancer Institute and Hospital, National Clinical Research Center of Cancer, Tianjin Key Laboratory of Cancer Prevention and Therapy, Tianjin, China; ^2^ State Key Laboratory of Pharmaceutical Biotechnology, School of Life Sciences, Nanjing University, Nanjing, China

**Keywords:** Bim, miR-24, pancreatic cancer, tumorigenesis, angiogenesis

## Abstract

miRNAs are a group of small RNAs that have been reported to play a key role at each stage of tumorigenesis and are believed to have future practical value. We now demonstrate that Bim, which stimulates cell apoptosis, is obviously down-regulated in pancreatic cancer (PaC) tissues and cell lines. And Bim-related miR-24 is significantly up-regulated in PaC. The repressed expression of Bim is proved to be a result of miR-24, thus promoting cell growth of both cancer and vascular cells, and accelerating vascular ring formation. By using mouse tumor model, we clearly showed that miR-24 promotes tumor growth and angiogenesis by suppressing Bim expression in vivo. Therefore, a new pathway comprising miR-24 and Bim can be used in the exploration of drug-target therapy of PaC.

## INTRODUCTION

Pancreatic cancer (PaC) is one of the common cancers worldwide, ranking forth in the causes of cancer-related deaths in western countries [1-3], and has the lowest survival rate (< 5%) among all the malignancies [3, 4]. A variety of studies have been focused on the novel anti-PaC agents, however, the direct molecular mechanism of cell growth, apoptosis and angiogenesis remains unclear.

Although it has been recognized that anti-apoptotic members of BCL-2 family (e.g., BCL2L1/BCL^−^xL) [5], inhibit autophagy and apoptosis of cancer cells, little known is about the biological function of pro-apoptotic members (e.g., BCL2L11/Bim, BID). Bim belongs to the pro-apoptotic BCL-2 family and is located in the outer mitochondrial membrane, where it acts as a central regulator of the intrinsic apoptotic cascade and mediates excitotoxic apoptosis [6]. The anti-apoptotic BCL2 members have multiple domains, while the pro-apoptotic members of BCL2 family are BH3-only proteins. But the role of Bim in cancers, especially in PaC, remains largely unexplored.

microRNAs (miRNAs) are a novel class of small non-coding RNAs which typically consist of 22 nucleotides [7]. So far, miRNAs have been found to be participated in various biological and pathological processes, including metabolism, cell growth and apoptosis, hormones secretion, aging, organic development and immune response [8, 9]. miRNAs are also known as a major regulator of the initiation and progression of human cancers, and our previous study demonstrated that serum miRNAs can be the potential biomarkers for the diagnosis of pancreatic cancer [10].

It has been reported that miR-24 is up-regulated in non-small cell lung cancer and leukemia and promotes the survival and proliferation of cancer cells [11, 12], and latest study reported that miR-24 was also involved in mesothelial cell integration of PaC [13]. In the present study, we found that miR-24 showed higher expression while Bim was significantly down-regulated in pancreatic tumor tissues. The subsequent bioinformatics analysis and luciferase assay demonstrated that Bim is a direct target of miR-24. Overexpression of miR-24 in PaC cells depressed Bim expression, thus accelerating cell proliferation, increasing the ratio of cells in S phase, and reduced cell apoptosis. In vascular endothelial cells, cell growth, apoptosis and ring formation were also proved to be regulated by miR-24 and Bim. We finally used tumor model mice to obtain the direct evidence that miR-24 promotes while Bim inhibits pancreatic tumor growth. To conclude, the miR-24-Bim pathway contributes to the complex network that activates pancreatic tumor growth and angiogenesis, and gives novel potential target for further therapy of PaC.

## RESULTS

### Bim is down-regulated in pancreatic cancer tissues

**Figure 1:**
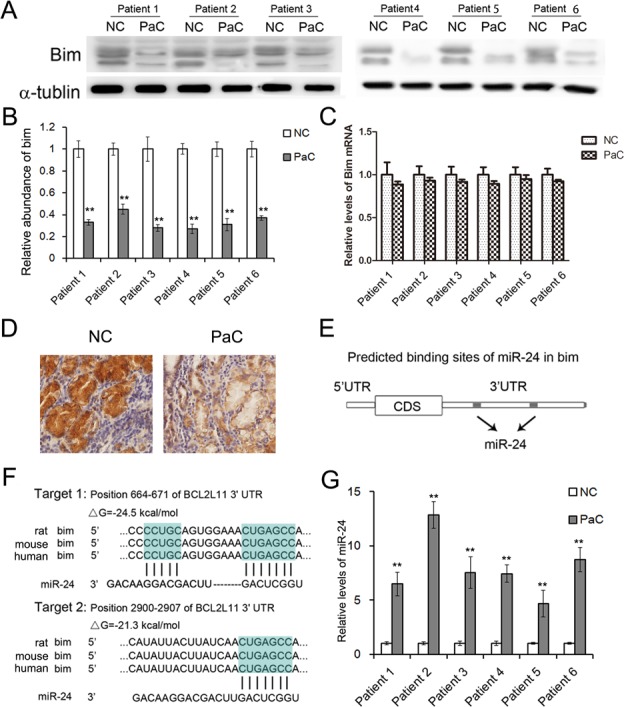
The expression patterns of Bim and miR-24 in PaC tissues **A.** Western blotting analysis of Bim protein levels in human PaC tissues (*n* = 6). **B.** Quantitative analysis of A. **C.** Relative levels of Bim mRNA in human PaC tissues (*n* = 6). **D.** Immunohistochemistry of the paraffin-embedded human pancreatic tumor tissues. **E.** The two predicted binding sites of miR-24 in the 3′UTR of Bim. **F.** Schematic description of the base-pairing interaction between miR-24 and Bim mRNA. **G.** Quantitative RT-PCR analysis of miR-24 levels in PaC tissues (*n* = 6). ** indicates *p* < 0.01.

Bim is known as an accelerator of cell apoptosis [14-16]. In this study, the expression of Bim in human PaC tissues was firstly determined by western blotting assay. Six pairs of PaC tissues and corresponding noncancerous tissues were collected, and Bim was found to be significantly down-regulated in PaC tissues (Figure 1A, 1B and 1D). The Bim protein was down-regulated by more than 60% on average, however, the mRNA level of Bim showed only slight decrease in PaC tissues compared to the noncancerous tissues (Figure 1C). We detected three isoforms of Bim with similar molecular weight (24 KD, 21 KD and 19 KD respectively). Bim is found to be mainly expressed in cancer cells instead of stroma cells by using IHC assays (Figure 1D). This result suggested that the expression of Bim mainly depends on the post-transcriptional regulation in PaC.

We also evaluated Bim expression pattern by using ‘Oncomine’ database, and it is showed that Bim is significantly down-regulated in pancreatic carcinoma (Supplemental Figure 1).

### Bim-related miR-24 is up-regulated in both PaC tissues and serum

We have previously reported a panel of miRNAs was dramatically changed in the serum of PaC patients and can be a potential diagnostic tool for early stage PaC [17]. Among the miRNAs, we found that miR-24, which is a predicted upstream regulator of Bim, was up-regulated in the serum of PaC patients (Supplementary Table 1). As is predicted, miR-24 directly binds two regions in 3′UTR of Bim mRNA (Figure 1E and 1F).

The miR-24 expression was measured by RT-qPCR, and it showed great increase in PaC tissues (Figure 1G). Thus miR-24 was selected for further experimental verification.

### miR-24 regulates Bim expression in PANC1 cells

**Figure 2:**
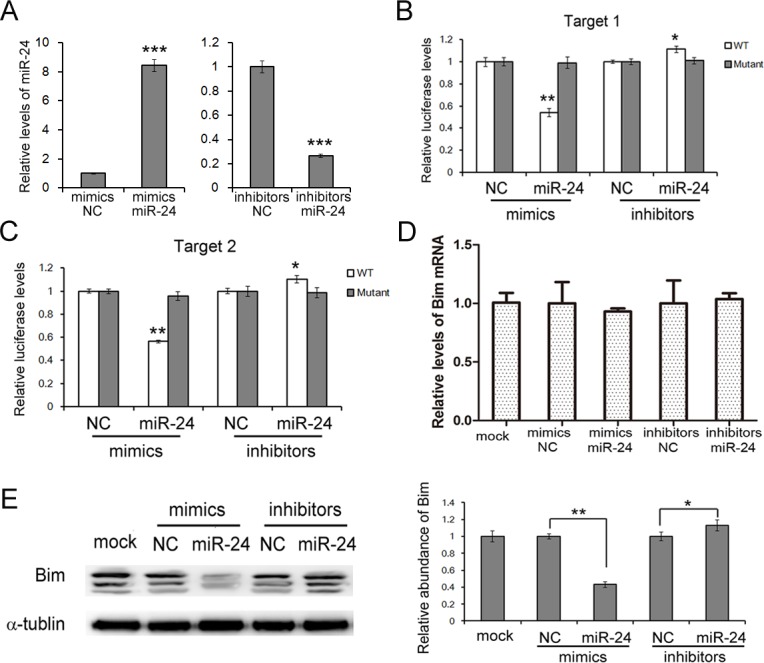
miR-24 suppresses Bim expression in PANC1 cells **A.** Relative levels of miR-24 in PANC1 cells transfected with mimics or inhibitors (*n* = 3). **B.** and **C.** Direct recognition of Bim 3′UTR by miR-24. PANC1 cells were co-transfected with firefly luciferase reporters containing either WT or mutant Bim 3′UTR with miR-24 mimics, inhibitors and the corresponding normal control. Cells were assayed using a luciferase assay kit at 24 h after transfection. The target 1 B.and target 2 **C.** of miR-24 were detected respectively (*n* = 3). **D.** and **E.** miR-24 suppresses Bim expression in PANC1 cells. Bim mRNA levels were assessed by quantitative RT-PCR **D.** and protein levels were analyzed by western blotting **E.** (*n* = 3). *** indicates *p <* 0.001; ** indicates *p <* 0.01; * indicates *p <* 0.05.

To give the direct evidence of the interaction between miR-24 and Bim, a luciferase assay was performed in HEK293T cells to evaluate the association. As is shown in Figure 2B and 2C, the relative luciferase activity was clearly inhibited when miR-24 mimics were co-transfected with the luciferase reporters containing one of the two predicted binding regions of the wild type (WT) 3′UTR of bim. However, the interaction was lost when the plasmid with a mutated sequence was used instead. And the co-transfection of miR-24 inhibitors and the plasmid with the WT Bim 3′UTR resulted in a relative increase in the luciferase signal (Figure 2B and 2C).

To study the biological role miR-24 in PaC cells, miR-24 mimics or inhibitors were used to interfere the expression of miR-24. Overexpression of miR-24 by mimics lead to a sharp reduction of Bim, while the inhibition of miR-24 slightly enhances Bim expression (Figure 2E). It is also clear that miR-24 does not affect Bim mRNA levels in PANC1 cells (Figure 2D).

These data demonstrated that miR-24 regulates Bim protein levels by directly binding two separated regions in Bim 3′UTR.

### miR-24 promotes cell proliferation while inhibits cell apoptosis of PANC1 cells

To assess the role of miR-24-Bim pathway in the process of cell growth, we used CCK8 kit to measure the growth rate of PANC1 cells [18].

**Figure 3:**
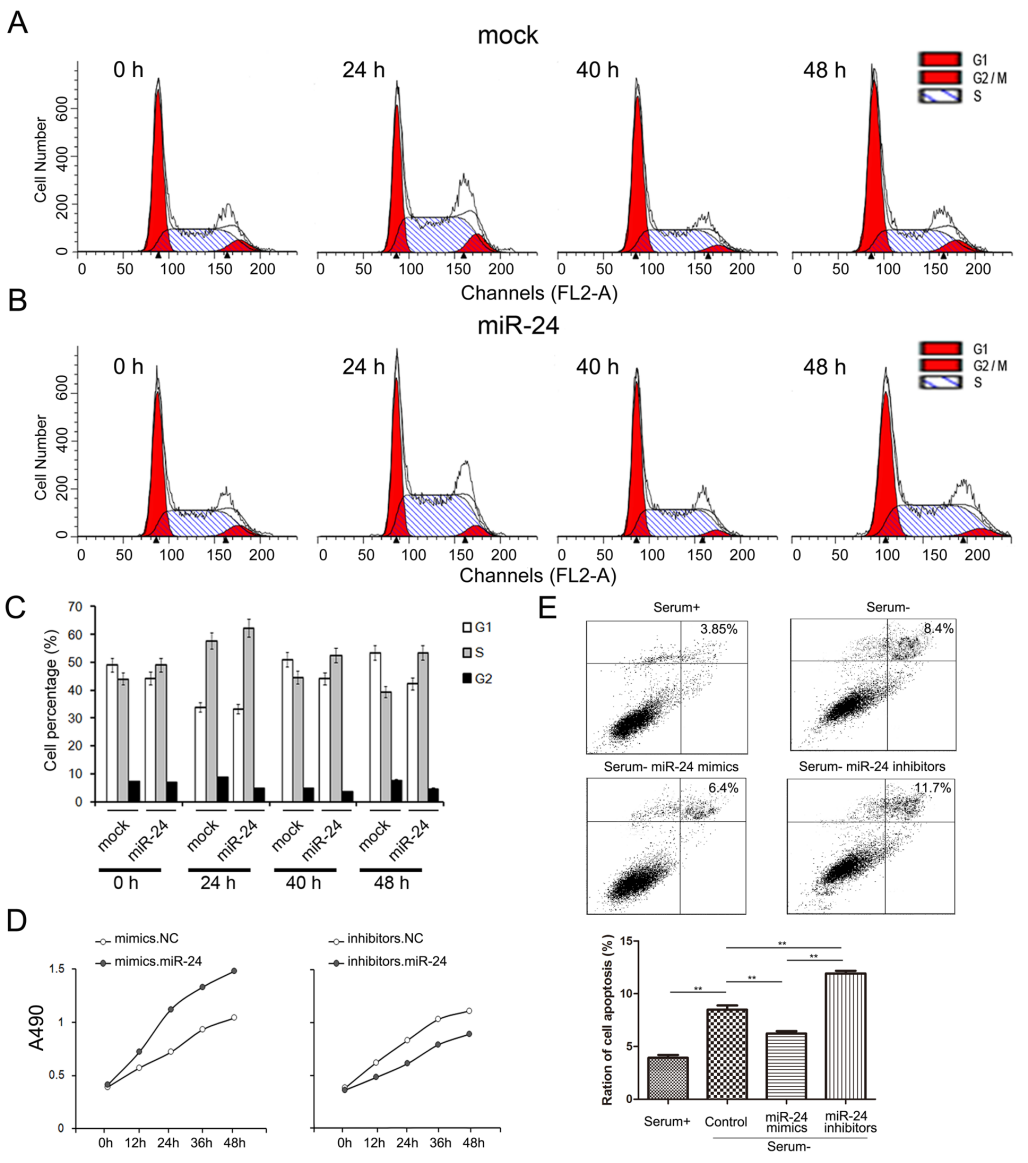
miR-24 promotes cell growth while inhibits apoptosis of PANC1 cells **A.** and **B.** miR-24 increases cell ratio in S phase. PANC1 cells were transfected with miR-24 mimics and cell cycle were evaluated by cell flow assay **B.** cells treated with water were used as normal control **A. C.** Quantified analysis of A and B (*n* = 5). **D.** miR-24 promotes cell proliferation of PANC1 cells (*n* = 5). **E.** miR-24 suppresses cell apoptosis (*n* = 5). ** indicates *p <* 0.01; * indicates *p <* 0.05.

The change of cell proliferation and apoptosis are mainly driven by cell cycle. In this study, the alteration of cell cycle was valued by cell flow assays. Following transfection, cells were harvested at o h, 24 h, 40 h and 48 h respectively. The cell ratios in S phase were clearly higher, while cell ratios in G1 phase were relatively decreased when miR-24 was up-regulated (Figure 3A, 3B and 3C).

Cell proliferation was evaluated by CCK8 assays. Overexpressed miR-24 strongly enhances while transfection of miR-24 inhibitors suppresses cell proliferation (Figure 3D).

Since the primary function of Bim is to accelerate cell apoptosis, we also determined the apoptosis levels of PANC1 cells using Annexin V-FITC/PI staining kit (BD Biosciences, CA, USA). As is expected, ratio of cell apoptosis significantly increased in FBS-free medium, and cell apoptosis was inhibited with the transfection of miR-24 mimics (Figure 3E).

It has been made clear that miR-24 plays an important role in regulating cell growth, apoptosis and cell cycle in PaC cell line.

### Knock-down of Bim boosts cell proliferation in PaC

**Figure 4:**
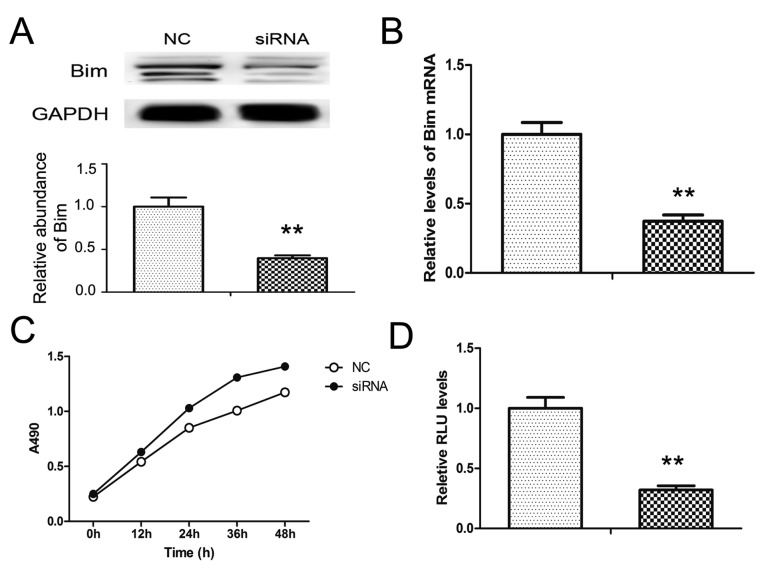
Knock-down of Bim in PANC1 cells Bim siRNA was transfected into PANC1 cells. **A.** and **B.** Bim protein **A.** and mRNA **B.** levels in PANC1 treated with Bim siRNA. **C.** and **D.** Effects of Bim siRNA on cell proliferation **C.** and apoptosis **D.** in PANC1 cells. *n* = 5; ** indicates *p <* 0.01.

To give a clear view of Bim-regulated cell growth and apoptosis, we used Bim siRNA to knock-down Bim protein in PANC1 cells. As is shown Figure 4A and 4B, both the mRNA and protein of Bim were markedly inhibited, and the Bim protein level was reduced by about 60% in PANC1 cells with the transfection of siRNA.

Subsequently, the function of Bim was also measured. As expected, cell proliferation was promoted while the cell apoptosis was repressed by Bim siRNA (Figure 4C and 4D). This result approved that Bim is an important regulator cell apoptosis and growth in PaC.

### Role of miR-24-Bim pathway in angiogenesis

**Figure 5:**
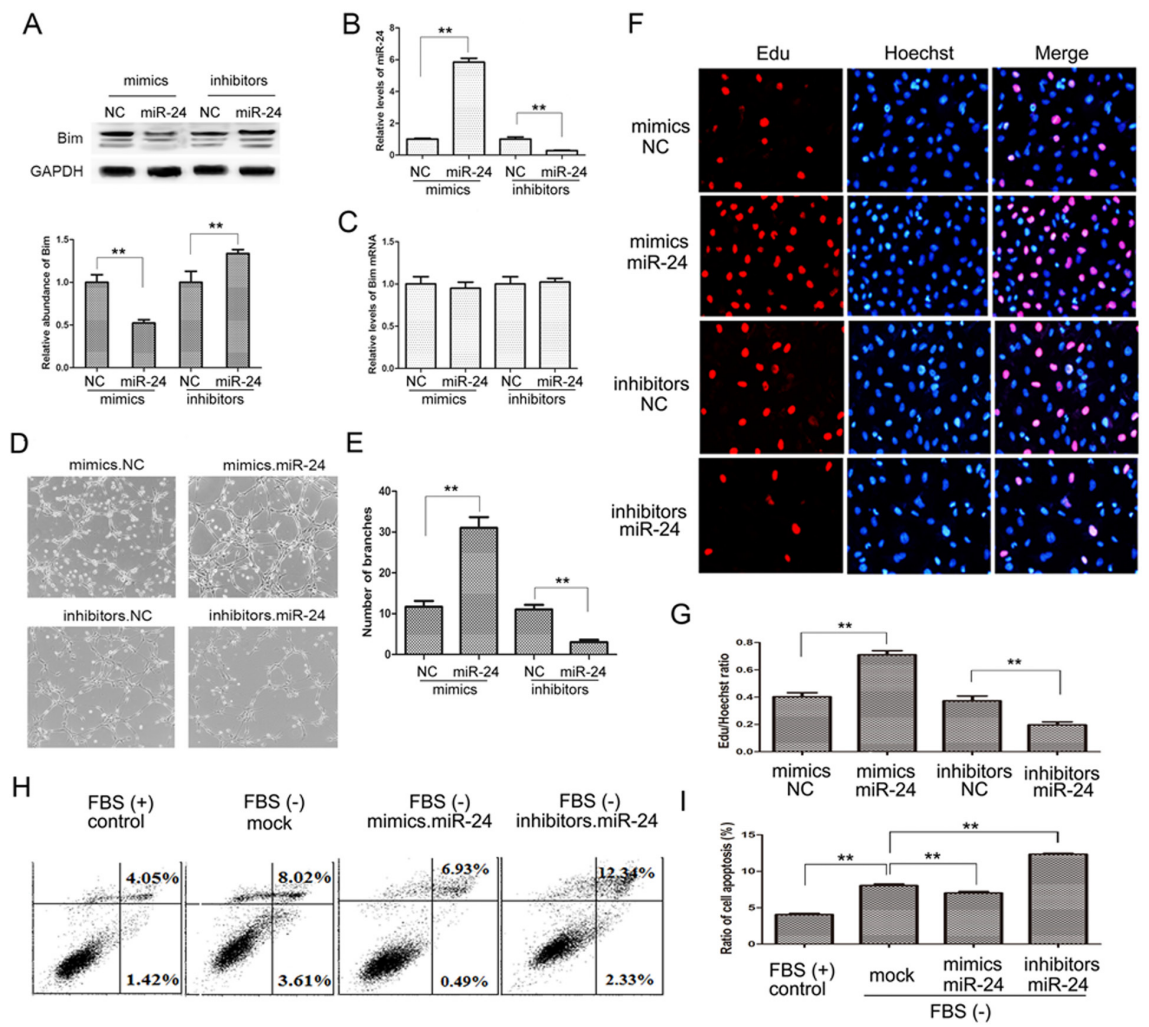
The miR-24-Bim pathway regulates angiogenesis **A.** and **C.** Bim protein **A.** and mRNA levels **C.** in HUVEC cells (*n* = 5). **B.** Relative levels of miR-24 in HUVEC cells treated with miR-24 mimics or inhibitors (*n* = 5). **D.** Representative images of HUVEC cells on Matrigel. **E.** Quantitative analysis of the experiments in panel D (*n* = 5). **F.** and **G.** Cell proliferation by performing an Edu incorporation assay (*n* = 5). **H.** and **I.** Cell apoptosis measured by flow cytometry (*n* = 5). ** indicates *p* < 0.01.

To find out the role of miR-24 in angiogenesis of pancreatic tumors, we checked vascular ring formation of HUVEC cells with the overexpression or knockdown of miR-24. As is shown in Figure 5A and 5B, Bim protein levels were reduced in HUVEC cells transfected with miR-24 mimics, while were enhanced by miR-24 inhibitors. However, the mRNA of Bim remained unchanged (Figure 5C). Subsequently, effects of miR-24 on angiogenesis were valued by endothelial tube formation assay. It is clear shown that overexpressed miR-24 promotes tube formation in HUVEC cells compared with control; whereas the tube formation was blocked as a result of miR-24 knockdown (Figure 5D and 5E).

Cell proliferation was valued by Edu/Dapi ration using immunofluorescence assays, and the cell growth rate increased by 80% with miR-24 mediated Bim inhibition; while cell growth rate was reduced by nearly 50% in miR-24 down-regulated cells compared with the control (Figure 5F and 5G).

The main biological function of Bim is to boost cell apoptosis, therefore, we also assessed apoptosis rate using cell flow assays. HUVEC cells were transfected with miR-24 mimics or inhibitors, and the medium was removed and fresh FBS-free RPMI-1640 was added to promote apoptosis. As is shown in Figure 5H and 5I, ratio of cell apoptosis clearly increased without FBS compared with control, and the apoptosis was inhibited in miR-24 overexpressed cells while was significantly enhanced in miR-24 down-regulated cells.

These data demonstrated that the miR-24-Bim pathway play a key role in regulating angiogenesis in PaC.

### miR-24 and Bim regulate tumor growth of PaC *in vivo*

**Figure 6:**
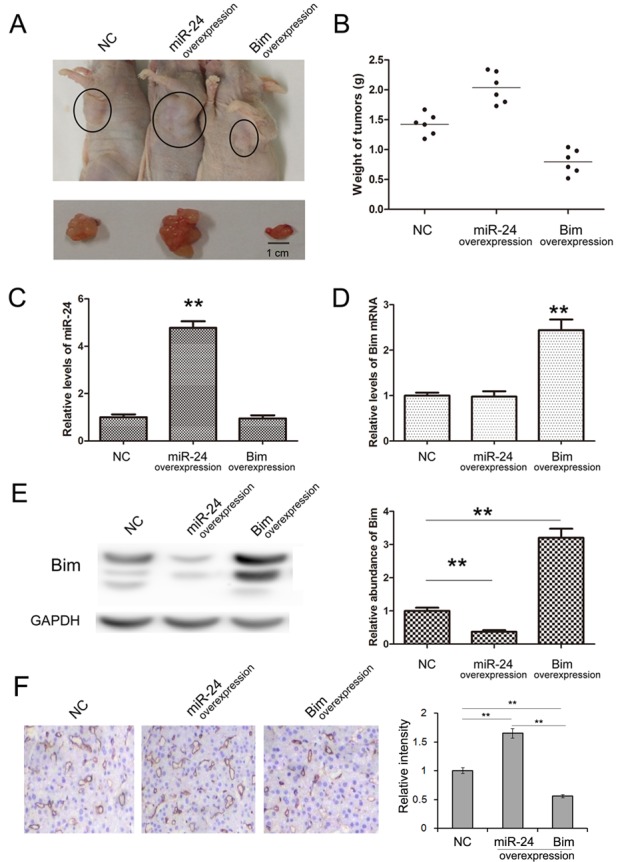
Effects of miR-24-Bim pathway on tumor growth *in vivo* **A.** Morphology of the tumors from tumor-implanted nude mice (*n* = 6). **B.** Weight of tumors excised from mice implanted with control PANC1 cells, miR-24-overexpressing PANC1 cells and Bim-overexpressing PANC1 cells. (*n* = 6) **C.** Relative levels of miR-24 in implanted tumor tissues (*n* = 6). **D.** and **E.** The Bim mRNA and protein levels in implanted tumor tissues (*n* = 6). **F.** Immunohistochemistry of the paraffin-embedded pancreatic tumor tissues shown in A (*n* = 6). ** indicates *p <* 0.01.

We also evaluate the function of miR-24 and Bim in the growth of human PaC cell xenografts in nude mice. The PANC1 cells were treated with lentivirus particles to rapidly produce high intracellular levels of mature miR-24 or Bim, and cells were harvested and injected subcutaneously in the armpit of mice. It is observed that the tumor sizes and weights are obviously increased in miR-24-overexpressing group compared with control, while the tumor growth is strongly inhibited in Bim-overexpressing group (Figure 6A and 6B). The expression of miR-24 and Bim in tumors was measured respectively. miR-24 raised by 4 folds and Bim raised by 3 folds in the corresponding overexpressing groups (Figure 6C, 6D and 6E).

To access the role of miR-24 and Bim in angiogenesis of PaC, IHC was performed using anti-CD31 antibody. As is shown in Figure 6F and 6G, CD31 expression was reduced in Bim-overexpression group; whereas overexpressed miR-24 increased CD31 expression. This result suggests that miR-24 promotes while Bim inhibits angiogenesis of PaC *in vivo*.

The tumor-implanted experiment offers a strong confirmation that miR-24-Bim pathway effectively regulates tumor growth in PaC, and implies that inhibition of miR-24 is a potential novel method for anti-PaC tumor.

## DISCUSSION

Dysregulation of miRNA expression in cancer has been widely reported in both tumor tissues and serum [19-22]. Tissue miRNAs provide an accurate diagnosis for various types of cancer, and usually lead to disorder of protein expression in cancer cells. In this study, Bim expression is found to be clearly repressed in tumor tissues of PaC, which is believed to contribute to the fast growth rate and reduced cell apoptosis of cancer cells.

We have previously reported the serum miRNA profile of PaC, and miR-24, which is among the predicted Bim-related miRNAs, was found to be up-regulated [10]. We further determined the miR-24 levels in PaC tumor tissues, and miR-24 expression is significantly increased. Concerning that Bim mRNA is consistent in PaC, miR-24 may be the main upstream regulator of Bim. The following experiments demonstrated that miR-24 directly targets Bim in both PaC cells and vascular cells, inhibiting cell apoptosis and promoting cell proliferation. The repression of Bim in vascular cells also accelerates ring formation. Therefore, the miR-24-Bim pathway is essential for tumor growth in both cancer cell proliferation and tumor angiogenesis. High expression of miR-24 in vascular cells may be secreted from PaC cancer cells, though it needs further evidence.

Over the past decades, chemotherapy and radiation have focused mass cell killing without specific targeting and often cause side effects and frequent failures [23-27]. Study on the molecular mechanism of tumor growth usually provides potential drug targets for future clinical application. In recent years, miRNAs or anti-miRNAs trafficked by microvesicles (MV) or plasmids have been used for the treatment of tumors and the other diseases in mouse models [28-30]. As far as we know, small RNAs can also be packed with nano-liposomes for in vivo-delivery and targeted therapy.

As is reported previously, miR-24 promotes cell proliferation in non-small cell lung cancer [31]; the high expression of miR-24 is associated with risk of relapse and poor survival in acute leukemia [12]; and the dysregulation of plasma miR-24 is also treated as clinical biomarker in nasopharyngeal carcinoma [32]. Based on these reports, we believe that miR-24 is a common oncogene among various types of cancer.

Taken together, we revealed an important miRNA-involved pathway that regulates tumor cell growth, apoptosis and tumor angiogenesis in PaC. It would therefore be of great interest to extend miR-24 to clinical research for the therapy of PaC as well as the other cancers.

## MATERIALS AND METHODS

### Animals

Male nude mice (BALB/c-nu, 6∼8 weeks) were purchased from the Model Animal Center of Nanjing University and were housed in a pathogen-free animal facility with access to water and food and allowed to eat and drink ad libitum. All of the experimental procedures were performed in accordance with protocols approved by the Institutional Animal Care and Research Advisory Committee of Nanjing University.

### Human tissue

Pancreatic cancer tissues and paired adjacent noncancerous tissues were derived from patients undergoing a surgical procedure at the Tianjin Medical University Cancer Institute and Hospital (Tianjin, China). Both tumor tissues and noncancerous tissues were confirmed histologically. The pathological type of each cancer was determined to be glandular carcinoma. Written consent was provided by all of the patients, and the Ethics Committee of Tianjin Medical University Cancer Institute and Hospital approved all aspects of this study. Tissue fragments were immediately frozen in liquid nitrogen at the time of surgery and stored at −80°C.

### Cell culture

Human pancreatic cell line PANC1, human embryo kidney epithelial cell line HEK293T were cultured in DMEM (Gibco, USA); and HUVEC was cultured in RPMI-1640 (Gibco, USA), both were supplemented with 10% fetal bovine serum (FBS, Gibco, USA) in a humidified incubator at 37°C with 5% CO_2_.

### RNA isolation and quantitative RT-PCR

Assays to quantify mature miRNAs were conducted as previously described with slight modifications [33, 34]. Total RNA was extracted from the cultured cells and tissues using TRIzol Reagent (Invitrogen) according to the manufacturer's instructions. miRNA determination was performed using Taqman microRNA probes (Applied Biosystems, Foster City, CA). All of the reactions were run in triplicate. After the reactions were complete, the cycle threshold (C_T_) data were determined using fixed threshold settings, and the mean C_T_ was determined from triplicate PCRs. A comparative C_T_ method was used to compare each condition to the control reactions. U6 snRNA was used as an internal control of miRNAs, and The mRNA levels of Bim was normalized to GAPDH. The relative amount of gene normalized to control was calculated with the equation 2^−ΔCT^, in which ΔC_T_ = C_T gene_-C_T_ control. Primers of Bim and GAPDH were as follows: 5′-AGAAGGCTGGGGCTCATTTG-3′ (GAPDH, sense); 5′-AGGGGCCATCCACAGTCTTC-3′(GAPDH, antisense); 5′-CACCAGCACCATAGAAGAA-3′ (Bim, sense); 5′-ATAAGGAGCAGGCACAGA-3′ (Bim, antisense).

### Cell transfection

Cells were seeded in a 6-well plate, and transfection was conducted after 24 h. The miR-24 overexpressing lentivirus, Bim overexpressing lentivirus and the control lentivirus were bought from GenePharma (Shanghai, China), and 10^6^ lentivirus were added into every single well with gentle mixing.

Cell transfection with miRNA mimics and inhibitors were conducted by using Lipofectamine 2000 (Invitrogen) according to the manufacturer's instructions. For each well, equal doses (100 pmol) of miRNA mimics, inhibitors, siRNAs (Santa Cruz, sc-29802), or scrambled negative control RNA were used. The cells were harvested at 24 h after transfection for real-time PCR analysis and western blotting.

### Lucifersase assay

Part of the wild type and mutated 3′UTR of Bim, containing the predicted miR-24 targeting regions, was synthesized and inserted into a p-MIR-report plasmid (Genescript, Nanjing, China). For luciferase reporter assays, 2 mg of firefly luciferase reporter plasmid, 2 mg of β-galactosidase expression vector (Ambion), and equal amounts (200 pmol) of mimics, inhibitors, or scrambled negative control RNA were transfected into cells. And the β-galactosidase vector was used as a transfection control. At 24 h after transfection, cells were assasyed using luciferase assay kit (Promega).

### Cell flow assays

For cell cycle analysis, cells were washed with phosphate-buffered saline solution (PBS) and fixed in 70% ethanol at 4°C for 2-4 h. After fixation, cells were washed twice with PBS before resuspension in propidium iodide/RNase A solution (5 μg/ml propidium iodide and 100 mg/ml RNase A). Cells were incubated with propidium iodide at room temperature in the dark for 1 h, then the stained cells were analyzed by flow cytometry.

For cell apoptosis analysis, cells were cultured overnight with both serum-containing complete medium and serum-depleted medium; the attached cells and floating cells were then harvested. Flow cytometry analysis of apoptotic cells was carried out using an Annexin V-FITC/PI staining kit (BD Biosciences, CA, USA). After washes with cold PBS, the cells were resuspended in binding buffer (100 mM HEPES, pH 7.4, 100 mM NaCl, and 25 mM CaCl_2_) followed by staining with Annexin V-FITC/PI at room temperature in darkness for 15 min. Apoptotic cells were then evaluated by gating PI and Annexin V-positive cells on a fluorescence-activated cell-sorting (FACS) flow cytometer (BD Biosciences, San Jose, CA). All experiments were performed in triplicate.

### Cell proliferation assay

PANC1 cells were collected 24 h, 48 h, 72 h, 96 h, 120 h, 144 h and 168 h post-transfection. After transfection, 10 μL of WST-8 was added into a corresponding test well and incubated for 4 h. Absorbance was measured at a wavelength of 450 nm.

### Immunofluorescence

HUVEC cells were incubated in 50 μM EdU (RiboBio Inc.) for 16 h, and fixed with 4% paraformaldehyde for 30 min at RT. Next, the cells were washed in PBS (2×5 min, RT) and then permeabilized using PBS containing 0.3% Triton X-100 for 10 min. After extensive washes in PBS, the cells were incubated in Apollo staining solution (RiboBio Inc.) for 20 min, washed with NaCl/Pi (3×10 min, RT), and then incubated in 4′,6-diamidino-2-phenylindole (DAPI, 1:2500; Roche Diagnostics, Mannheim, Germany) for 10 min at RT.

### Angiogenesis *in vitro*

The *in vitro* endothelial tube formation assay was performed as previously described [29, 35, 36]. Briefly, 100 μl of Matrigel (BD Bioscience) was added to each well of a 24-well plate and allowed to polymerize at 37°C for 30 min. HUVEC cells were firstly transfected with miR-24 mimics or inhibitors, and subsequently, cells were resuspended in FBS-free 1640 medium and seeded in each well at a concentration of 1 × 10^5^ cells/well. After 6 h, the cells were examined under a light microscope to assess the formation of capillary-like structures. The branch points of the formed tubes, which represent the degree of angiogenesis *in vitro*, were scanned and quantified in five low-power fields (200×).

### Immunohistochemistry

The tumors were fixed in 4% paraformaldehyde, embedded in paraffin, sectioned, and then stained with DBE-conjugated anti-CD31 antibodies (Abcam, USA) and DBE-conjugated anti-Bim antibodies (Santa Cruz, USA). Quantitative analysis was conducted by quantifying the fluorescence intensity from six sections.

### Establishment of tumor Xenograft in nude mice

PANC1 cells treated with control lentivirus or miR-24 overexpressing lentivirus or Bim overexpressing lentivirus were injected subcutaneously into nude mice (1×10^7^ cells for one mouse). Mice were sacrificed after 4 weeks, and the weight and diameter of tumors were recorded.

### The miRNA target prediction

The miRNA target prediction and analysis were performed with the algorithms from TargetScan (http://www.targetscan.org/) PicTar (http://pictar.mdc-berlin.de/) and miRanda (http://www.microrna.org/).

### Western blotting analysis

The Bim expression was assessed by western blotting analysis and samples were normalized to GAPDH. Protein extraction was blocked with PBS-5% fat-free dried milk at room temperature for 1 h and incubated at 4°C overnight with anti-Bim (1:500, Santa Cruz), and anti-GAPDH (1:2000, Santa Cruz) antibodies respectively.

### Statistical analyses

All data were representative of five or six independent experiments. Data were expressed as mean ± S.E. of at least five separate experiments. Statistical significance was considered at *P* < 0.05 using the Student's *t*-test. In this study, ‘*’ indicates ‘*P* < 0.05’, ‘**’ indicates ‘*P* < 0.01’, and ‘***’ indicates ‘*P* < 0.001’.

## SUPPLEMENTARY MATERIAL FIGURE AND TABLE


