# Supplementary material for: The miR-24-Bim pathway promotes tumor growth and angiogenesis in pancreatic carcinoma
Source: Oncotarget. 2015 Oct 28;6(41):43831–42. doi: 10.18632/oncotarget.6257 (PMC4791270; doi:10.18632/oncotarget.6257)
Supplement: Supplementary file 1 [file oncotarget-06-43831-s001.pdf]

The miR-24-Bim pathway promotes tumor growth and angiogenesis in pancreatic carcinoma

Supplementary Material

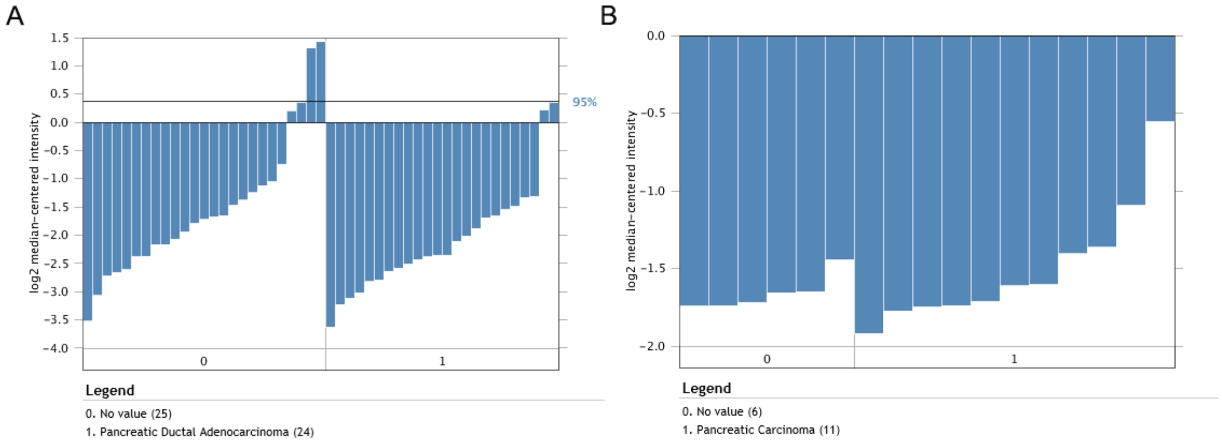

Supplementary Figure 1: Bim expression pattern in PaC tissue. A. BCL2L11 Expression in pancreatic ductal adenocarcinoma (n=49). **(**Cancer Sci 2005/07/01). B. BCL2L11 expression pancreatic carcinoma (n=17). (Clin Cancer Res 2005/05/01).

Supplementary Table 1: Differentially-expressed miRNAs in PaC serum samples compared to those in control serum samples determined by sequencing technology.

order	name	mean fold change	p-value	
1	let-7a	1.0943746	0.586468	non-significant
2	let-7b	0.978667	1.6789	non-significant
3	let-7c	1.789553	1.789564	non-significant
4	let-7d			undetectable
5	let-7f			undetectable
6	let-7i			undetectable
7	miR-1			undetectable
8	miR-20a	3.13	3.64E-07	significant
9	miR-21	4.24	2.28E-05	significant
10	miR-22			undetectable
11	miR-24	2.67	0.001255	significant
12	miR-25	5.08	8.18E-10	significant
13	miR-26a	4.16	0.128493	non-significant
14	miR-27a	1.2960753	0.008085	non-significant
15	miR-27b	1.07	0.7567261	non-significant
16	miR-28-3p			Cq value > 35
17	miR-29a	1.1431785	0.3623874	non-significant
18	miR-29c	1.320792	0.1492103	non-significant
19	miR-30a	1.4954582	0.0731187	non-significant
20	miR-30d	0.8956235	0.5688125	non-significant
21	miR-92a	1.2414502	0.3052584	non-significant
22	miR-95	1.1179528	0.5932393	non-significant
23	miR-99a	2.69	2.61E-05	significant
24	miR-100	1.0234521	0.9108538	non-significant
25	miR-101	1.079218	0.7229841	non-significant
26	miR-103	1.0661197	0.7972754	non-

				significant
27	miR-107	1.094375	0.586468	non-significant
28	miR-122	3.26	0.104824	non-significant
29	miR-125a-5p			undetectable
30	miR-125b	1.3597159	0.2975648	non-significant
31	miR-128	1.0538031	0.8654724	non-significant
32	miR-139-3p	1.053803	0.865472	non-significant
33	miR-140-3p	1.042287	0.7037122	non-significant
34	miR-146-5p			undetectable
35	miR-148a	1.0481299	0.8971156	non-significant
36	miR-150	1.746494	0.057758	non-significant
37	miR-185	2.1	0.007051	significant
38	miR-185*			undetectable
39	miR-191	2.87	0.000372	significant
40	miR-192	1.3246894	0.2644458	non-significant
41	miR-193b*			Cq value > 35
42	miR-197	1.117953	0.593239	non-significant
43	miR-199a-3p	1.363585	0.2153148	non-significant
44	miR-206			undetectable
45	miR-210	1.3872444	0.3868377	non-significant
46	miR-215	0.9732919	0.9444299	non-significant
47	miR-221			undetectable
48	miR-222	0.9764046	0.8690469	non-significant
49	miR-320a	1.493328	0.0640379	non-significant
50	miR-320b			Cq value > 35
51	miR-339-5p			undetectable
52	miR-361	1.5194114	0.3374292	non-

				significant
53	miR-378	1.2340253	0.4139875	non-significant
54	miR-411	1.796262	0.007805	non-significant
55	miR-423-5p			undetectable
56	miR-484			Cq value > 35
57	miR-451	1.7464941	0.0577575	non-significant
58	miR-483	5.632765	0.1209546	non-significant
59	miR-486-3p			undetectable
60	miR-486-5p			undetectable
61	miR-532-5p	1.7962625	0.0078049	non-significant
62	miR-532-3p			undetectable
63	miR-584			Cq value > 35
